# Alternative Polyadenylation Signatures Distinguish Maladaptive Right Ventricular Remodeling in Pulmonary Hypertension: Implications for RNA-Based Diagnostics and Therapeutics

**DOI:** 10.3389/bjbs.2026.15687

**Published:** 2026-02-20

**Authors:** Janani Subramaniam, Venkata Jonnakuti, Scott D. Collum, Sandra Martineau, Kai-Lieh Huang, Sandra Breuils-Bonnet, Andrea L. Frump, Bindu H. Akkanti, Jayeshkumar A. Patel, Manish K. Patel, Ismael Salas de Armas, Isabella N. Lefebvre, Rajko Radovancevic, Elvin Blanco, Eric J. Wagner, Igor Gregoric, Sriram Nathan, Biswajit Kar, Steeve Provencher, Sebastien Bonnet, François Potus, Hari Krishna Yalamanchili, Harry Karmouty-Quintana

**Affiliations:** 1 Division of Pulmonary, Critical Care and Sleep Medicine, Department of Internal Medicine, McGovern Medical School, University of Texas Health Science Center at Houston, Houston, TX, United States; 2 Jan and Dan Duncan Neurological Research Institute, Texas Children’s Hospital, Houston, TX, United States; 3 Centre de Recherche de l’Institut Universitaire de Cardiologie et de Pneumologie de Québec (IUCPQ), Université de Laval, Québec, QC, Canada; 4 Department of Biochemistry and Biophysics, University of Rochester Medical Center, Rochester, NY, United States; 5 Department of Medicine, Indiana University School of Medicine, Indianapolis, IN, United States; 6 Center for Advanced Cardiopulmonary Therapies and Transplantation, McGovern Medical School, University of Texas Health Science Center at Houston, Houston, TX, United States; 7 Department of Nanomedicine, Houston Methodist Research Institute, Houston, TX, United States; 8 USDA/ARS Children’s Nutrition Research Center, Department of Pediatrics, Baylor College of Medicine, Houston, TX, United States

**Keywords:** 3’UTR shortening, CPSF5, decompensated RV, right heart failure, right ventricle

## Abstract

Increased pulmonary vascular pressures due to vascular remodeling, elevated vascular resistance, and vasoconstriction characterize Pulmonary Arterial Hypertension (PAH). The narrowing of the pulmonary arteries and obstruction of blood flow increase the Right Ventricular (RV) afterload, forcing the RV to undergo structural and functional changes. While adaptive remodeling leads to RV compensation by maintaining function, maladaptive remodeling leads to RV decompensation, characterized by worsening function and eventual failure. At present, there is no effective treatment for these patients as therapies for left ventricular failure are ineffectual, and there are no therapies specifically targeting the RV. Therefore, there is a clear need to understand the pathophysiology of RV failure and to identify the differences between adaptive and maladaptive RV remodeling. This study analyzes changes in polyadenylation site usage, a process known as alternative polyadenylation (APA), in RV failure. APA is a mechanism used to regulate mRNA maturation that can result in either shortening or elongation of the mRNA 3’UTR. By analyzing APA patterns in RV tissue from donor controls and patients with compensated and decompensated RV failure, we demonstrate a pattern of 3’UTR elongation that is present in decompensated RV failure and not in compensated or control RVs. Further, altered APA was also detected in 3 distinct rat models of PH, where 15 transcripts had shared APA alterations across both rat models and human disease. Our study provides an unbiased approach to identifying the molecular changes leading to RV dysfunction while pinpointing novel therapeutic targets that can be leveraged for intervention. These APA signatures may serve as biomarkers to distinguish adaptive from maladaptive RV remodeling. In addition, the RNA-processing machinery that regulates APA, such as NUDT21 and CPSF6, represents potential therapeutic targets for RNA-based interventions. Together, our findings link RNA processing to diagnostic and therapeutic opportunities in right heart failure.

## Graphical Abstract

**Figure:**
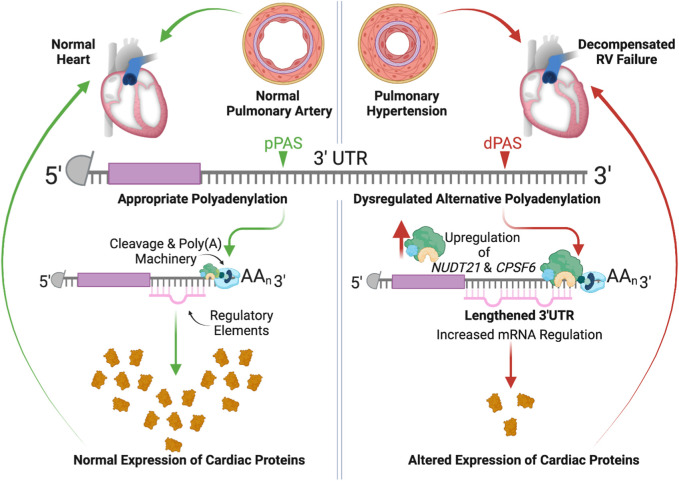
In the healthy state, CPA appropriately recognizes PAS, supporting normal protein expression. In PH, dysregulated APA, driven by factors like *NUDT21* and *CPSF6*, cause lengthened 3'UTRs and altered protein expression. This disrupts cardiac function, contributing to maladaptive remodeling and right ventricular failure, highlighting APA’s role in disease progression.

## Introduction

Right ventricular (RV) function is a key determinant of prognosis in pulmonary arterial hypertension (PAH) [1]. Patients with stable or improving RV function have 5-year survival rates exceeding 90%, while those with progressive RV dysfunction have survival below 30% [2]. Unlike left ventricular (LV) failure, RV failure lacks effective targeted therapies, and many treatments beneficial in LV dysfunction are ineffective or contraindicated in RV disease [3]. Although carvedilol, macitentan, and sotatercept have shown potential benefits [4–6]; none directly target RV-specific mechanisms, highlighting an urgent need for additional mechanistic understanding.

RV failure in PAH arises primarily from increased afterload due to pulmonary vascular remodeling [7]. While the RV initially compensates through hypertrophy and remodeling, persistent stress leads to maladaptation, dilation, and failure [8–10]. This transition strongly predicts morbidity and mortality, underscoring the importance of defining the molecular pathways underlying RV remodeling. Prior studies have identified drivers of maladaptive RV remodeling, including apoptosis, capillary rarefaction, metabolic reprogramming, oxidative stress, neurohormonal activation, inflammation, and fibrosis [11–17].

Recent transcriptomic and proteomic analyses have further characterized these processes, revealing complex regulatory networks involved in RV remodeling and decompensation [18–23]. However, the mechanisms driving widespread gene expression changes in failing RVs remain incompletely understood. One promising avenue involves RNA processing defects, which are increasingly recognized as both biomarkers and therapeutic targets across disease contexts. RNA-based technologies such as antisense oligonucleotides and lipid nanoparticles have already demonstrated clinical utility, and similar approaches could be harnessed to target APA regulators in right ventricular failure.

Polyadenylation is a fundamental step in mRNA maturation in eukaryotes, involving endonucleolytic cleavage of the nascent pre-mRNA and addition of a poly(A) tail at its 3′ end, which promotes mRNA stability, export, and translation [24]. The use of multiple polyadenylation sites within the same transcript allows alternative polyadenylation (APA), generating distinct 3′-end isoforms that can differ in untranslated region (3′UTR) length and regulatory potential [25].

Alternative polyadenylation (APA) is a post-transcriptional regulatory process that generates mRNA isoforms with variable 3′ untranslated region (3′UTR) lengths, influencing transcript stability, localization, and translation [26, 27]. APA is tightly regulated by the cleavage and polyadenylation (CPA) machinery, which includes CPSF, CstF, CFIm, and CFIIm complexes [28, 29]. Dysregulated APA contributes to multiple pathologies, including cancer, fibrosis, and maladaptive cardiac remodeling [25, 30–38]. For example, NUDT21 downregulation induces global 3′UTR shortening and oncogene upregulation in glioblastoma [32], while loss of NUDT21 in pulmonary fibrosis drives shortened 3′UTRs of profibrotic genes such as *COL1A* and *TGFBR1* [39] and is associated with increased *HAS2* expression in PAH(36). In contrast, 3′UTR lengthening is associated with neuronal differentiation, senescence, and altered stress responses [40–42]. For these reasons, this study has focused on the assessment of alterations in the 3’UTR in RV dysfunction, where similar to other disease states we have identified a unique dysregulation. However, it is important to note that additional RNA regulatory mechanisms may play a pathophysiological role such as alterations in translation efficiency [43]. Importantly, APA exists alongside other post-transcriptional processes such as alternative splicing, mRNA stability control, translational regulation, and noncoding RNA mediation, all of which contribute to the diversity and regulation of gene expression [44].

Given the role of APA in diseases that share pathophysiologic features with RV failure, we hypothesized that APA dysregulation contributes to the progression of RV dysfunction. Here, we analyzed APA patterns in human RV tissue from donor controls and patients with compensated and decompensated RV failure, alongside three rat models of PAH. We identified significant disease-associated APA alterations, including 3′UTR lengthening unique to decompensated RVs and a set of conserved transcripts dysregulated across species. These findings provide new insight into post-transcriptional mechanisms driving RV failure and highlight potential targets for therapeutic intervention.

## Methods

### Ethics

All human tissues were obtained with informed consent. We utilized RNA-seq data publicly available from the Gene Expression Omnibus (GEO) database (accession number: GSE198618). Samples used for RT-qPCR and western blot validation were obtained by the Pulmonary Center of Excellence biorepository at UTHealth Houston following the institutional review board (IRB) approval (HSC-MS-08-0354 and HSC-MS-15-1049). All animal protocols were approved by the Institutional Animal Care and Use Committee (IACUC) according to the guidelines established by the international Association for Assessment and Accreditation of Laboratory Animal Care (AAALAC, “Guide for the Care and Use of Laboratory Animals,” National Academies Press 2011). The rodents were euthanized in a carbon dioxide chamber followed by cervical dislocation and bilateral thoracotomy in accordance with protocols established by the American Veterinary Medical Association (AVMA POE 2020 Guidelines).

### Human RV Data

Gene expression data used in this study was obtained from the Gene Expression Omnibus (GEO) database (accession number: GSE198618). The dataset includes RNA-sequencing data from RV tissues of human subjects clinically categorized into three groups: compensated RV function (n = 11), decompensated RV function (n = 7), and donor controls (n = 14). A compensated RV is defined by a state in which the RV maintains adequate cardiac output despite increased pressure or volume load, through adaptive mechanisms such as hypertrophy, enhanced contractility, or augmented preload, without overt signs of systemic congestion or low perfusion. A decompensated RV is defined by a condition characterized by the inability of the RV to sustain effective forward flow, resulting in elevated right-sided filling pressures, systemic venous congestion, and impaired end-organ perfusion [45].

The dataset is publicly available through the GEO database at https://www.ncbi.nlm.nih.gov/geo/query/acc.cgi?acc=GSE198618.

### PAC-Seq Library Preparation and Sequencing

Total RNAs from human and rat heart tissue were extracted using Trizol (Invitrogen), and1 µg of RNA was harvested. This RNA was subsequently reverse transcribed with a partial p7 adaptor (Illumina_4N_21T: GTGACTGGAGTTCAGACGTGTGCTCTTCCGATCTNNNNTTT TTT​TTT​TTT​TTT​TTT​TTT) and dNTPs with the additional of spiked-in azido-nucleotides (AzVTPs) at a 5:1 ratio. Subsequently, we utilized copper(I)-catalyzed azide-alkyne cycloaddition (CuAAC) to click the P5 adaptor (5′Hexynyl-NNNNAGATCGGAAGAGCGTCGTGTAGGGAAAGAGTGTAGAT CTC​GGT​GGT​CGC​CGT​ATC​ATT, IDT) to the 5′ end of the cDNA. We next amplified the libraries with a universal primer (AAT​GAT​ACG​GCG​ACC​ACC​GAG) and 3′indexing primer (i.e., Index 1: CAA​GCA​GAA​GAC​GGC​ATA​CGA​GAT​CGT​GAT​GTG​ACT​GGA​GTT​CAG​A CGTGT). These libraries were then purified on a 2% agarose gel at 200–300 bps molecular sizes. Purified libraries were pooled and sequenced by Illumina NextSeq 500/550 High Output Kit v2.5 (150 cycles) (Cat# 20024907).

### Bioinformatic Analyses

Differential expression analyses were performed with DESeq2 (v1.36.0), as described in [46], a variance-analysis package developed to infer statistically significant differences in RNA-seq data using a Negative Binomial GLM. Genes are called differentially expressed (DE) when having a Fold-Change (FC) >0.263 or <−0.263, and FDR <0.05 (using Benjamini-Hochberg method to control false discovery rate). For the differential expression analysis of the human data, raw count matrices were directly downloaded from the GEO database (GSE198618). APA dynamics from bull RNA-Seq data were analyzed using PolyAMiner-Bulk [47]. Data was processed with the following parameters: -a 0.65 -pa_p 1 -pa_a 5 -pa_m 5 -outPrefix 3UTROnly -expNovel 1 -s 2 - ignore UTR5, CDS, Intron, UN -apriori_annotations -modelOrganism human. PAC-Seq data were analyzed using PolyA-miner [48]. Read counts for each Cleavage and (PAS) were calculated as the total number of reads mapped to the site. Total gene counts were then determined as the sum of all C/PAS counts across the individual features within the gene. PolyAMiner-Bulk is adept at inferring changes in alternative polyadenylation (APA) from bulk RNA-Seq datasets. It enhances the detection process by integrating both established reference polyadenylation sites and newly detected C/PASs. It further refines the candidate C/PASs by applying a deep learning model called C/PAS BERT. PolyA-miner uses vector projections and beta-binomial test to analyze APA patterns within genes. To perform the Gene Ontology (GO) enrichment analysis, we used the gprofiler2 R package to query a list of genes against the Biological Process (GO:BP) database. Results were filtered by an adjusted *p*-value cutoff (≤0.05) and visualized.

### Rodent PH Models

MCT model: For the animal studies, male and female Sprague Dawley rats (*Rattus norvegicus*) at age 8–12 weeks (Charles River Laboratories) were subjected to the treatment protocol as previously described [49]. In brief, for the MCT model, a single subcutaneous injection of MCT (60 mg per kg body weight; n = 4) was applied (control rats received saline; n = 4), and the RV function was monitored. The whole experiment procedure was 6 weeks for the MCT rat model. For the PAB model, following anesthesia, the PA was separated from the aorta and left atrium, was tied against a 19-gauge needle and then released quickly, as previously described [49]. PAB-operated rats (n = 4) and sham-operated rats (n = 4) without tying the pulmonary trunk were included for the study. Rats were studied between weeks 3 and 8 following PAB operation. Finally, for the hypoxia/Sugen model, rats received a single IP injection of Sugen5416 (20 mg/kg) followed by exposure to 10% oxygen for 3 weeks and returned to normoxia for an additional 2 weeks. Rats housed in normoxia conditions throughout (n = 5) and subjected to SUHx (n = 4) were assessed for PH. For right heart catheterization, rats were anesthetized with continuous inhalation of 1.5%–2.0% isoflurane under a nosecone.

### RNA Isolation and RT-qPCR

Flash frozen RVs were pulverized with a mortar and pestle. RNA was extracted with Triazole and chloroform. RNA was obtained by addition of Cotrimoxazole lysis reagent (QIAzole, QIAGEN; Hilden, Germany) followed by extraction with chloroform added in a 1:5 ratio. Following precipitation with 100% ethanol, samples were passed through an RNA-binding spin column (miRNeasy QIAGEN). The column was washed and mRNA eluted with water. Total RNA was quantified by A260/280 ratio via NanoDrop. cDNA synthesis was performed by reverse transcription reaction (iScript, BioRad) using 1000 ng of template. Fold change against the housekeeping gene *18S* was determined via the double delta C_t_ method. Fluorometric amplification signal was monitored in real time with an ABI 7500 Real-time PCR system (Applied Biosystems; Foster City, CA).

### Immunoblotting

Pulverized heart tissue was dissolved in RIPA buffer (Thermo Scientific, Rockford, IL) containing 1 mM protease and phosphatase inhibitor (Sigma-Aldrich). 30 mcg of protein was mixed with 6X SDS-Sample buffer (Boston BioProducts) and loaded into 4%-12% Mini-Protean TGX gels (Bio-Rad, Hercules, CA). These gels were then transferred to PVDF membranes (GE Healthcare, Piscataway, NJ), which were subsequently blocked with 5% milk in TBS-T(Sigma-Aldrich) for 1 h and incubated with the primary antibody overnight at 4 °C. Secondary antibodies were incubated for 1 h at RT and the blots were visualized with Clarity ECL (Bio-Rad). Densitometries were performed with the Bio-Rad Image Lab software.

## Results

### Altered 3’UTR Landscape and Gene Expression Are Present in Decompensated but Not Compensated RV

**FIGURE 1:**
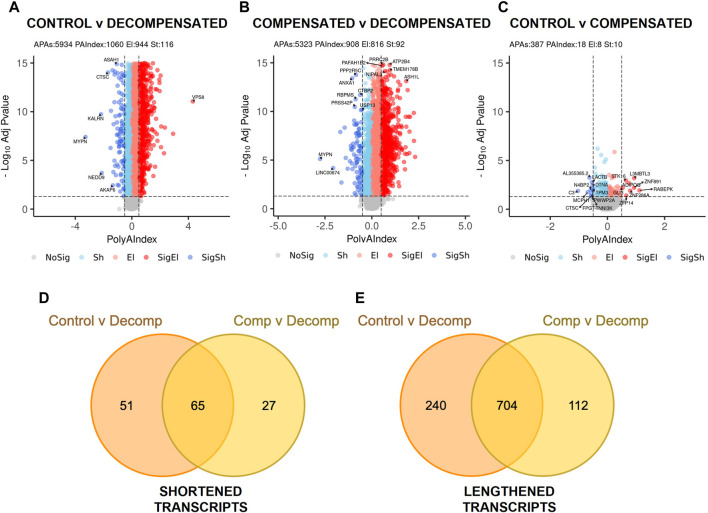
APA drives disease progression in RV decompensation. Volcano plots illustrate transcript length alterations in **(A)** Control vs. Decompensated, **(B)** Compensated vs. Decompensated, and **(C)** Control vs. Compensated RVs, where dark red = significantly elongated (SigEl), light red = elongated (El), dark blue = significantly shortened (SigSh), light blue = shortened (Sh) and grey = unchanged (NoSig) transcripts. The horizontal dashed lines indicate the −log_10_ (*P* adjusted) ≥1.325 (*P* adjusted ≤0.05), and the vertical dashed lines indicate polyA index ≥+0.1 and ≤−0.1, with positive values indicating longer transcripts and negative values indicating shorter transcripts. Venn diagrams show the overlap of **(D)** APA shortening events and **(E)** lengthening events, driving the decompensation phenotype.

**FIGURE 2:**
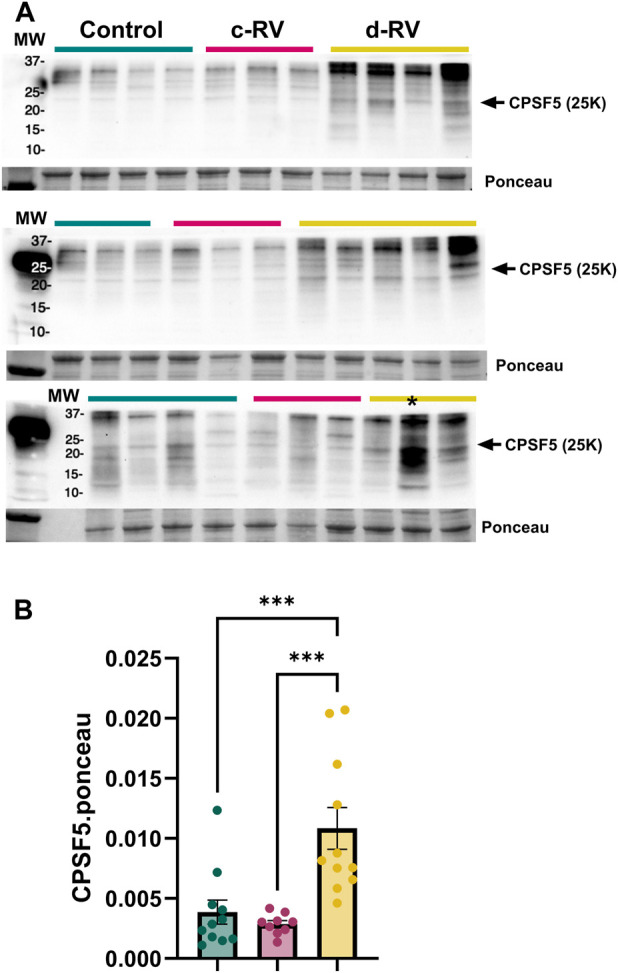
Increased expression of Cleavage and Polyadenylation Specificity Factor (CPSF) 5 in the decompensated right ventricles (RV). **(A)** Western blots for CPSF5 and control ponceau stain performed from isolated control (N = 11, teal), compensated (N = 9, pink) and decompensated RVs (N = 12, yellow). Quantification data from each individual WB denoting CPSF5 levels *denotes outlier excluded from quantification **(B)**. Significance levels ***p˂0.001 refer to comparisons between control or compensated RV vs. decompensated RV.

We detected a significant change in the 3’UTR landscape and APA genes (DAGs) in decompensated RVs compared to control RVs (Figure 1A) that was more pronounced in comparisons between decompensated RVs compared to compensated RVs (Figure 1B), reflecting minimal changes in gene regulation between compensated RVs and controls. Only 18 significantly differentially regulated genes were observed in compensated RVs (Figure 1C). 3’UTR landscape of decompensated RVs reflected 3’UTR elongation as the predominant shift in APA, with 944 transcripts with longer 3’UTRs and 116 mRNAs with shorter 3’UTRs relative to control RVs (Figure 1A). Comparisons between compensated vs. decompensated RVs yielded 816 significant transcripts with longer 3’UTRs and 92 transcripts with shorter 3’UTRs compared to compensated RVs (Figure 1B). Comparisons between controls vs. decompensated and compensated vs. decompensated revealed 65 common genes that were shortened (Figure 1D) and 704 elongated transcripts (Figure 1E). Thus, global 3'UTR lengthening appeared specific to RV decompensation and may be a distinctive marker for progressed RV disease. Previous studies have identified, 3’UTR shortening and concomitant reduced expression of CPSF5 [36, 39, 50] as a pathological mechanism. In line with this, we aimed to assess expression levels of CPSF5 in decompensated human RVs compared to compensated RVs. Herein we report increased CPSF5 levels, consistent with 3’UTR elongation (Figures 2A,B). This is significant since 3’UTR shortening is often associated with loss of CPSF5(39) and an increase of this RNA binding protein may imply 3’UTR elongation. A complete list of APA analyses is provided in Supplementary Table S1.

Enrichment analyses comparisons between control RV Vs. decompensated RV and compensated RV vs. decompensated RV revealed enrichment of several key processes, including the Longevity Regulating pathway, AMP-activated protein kinase (*AMPK*), p53, insulin, Forkhead box O (*FoxO*) signaling pathways and lysine degradation (Supplementary Figures S1A, B). Pathways that were unique to control RV vs. decompensated RV included cellular senescence, human papillomavirus infection, phosphatidylinositol 3-kinase (PI3K-Akt) and pathways in cancer (Supplementary Figure S1A). Insulin resistance, HIF-1A, adiponectin signaling ad Mannose Type O-glycan synthesis were unique to compensated RV vs. decompensated RV comparisons (Supplementary Figure S1B).

**FIGURE 3:**
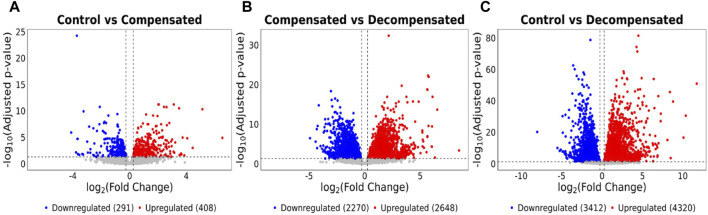
Transcriptomic analysis reveals severe gene expression dysregulation in diseased RVs. Volcano plots of **(A)** Control vs. Decompensated, **(B)** Compensated vs. Decompensated, and **(C)** Control vs. Compensated RVs, identify DEGs in healthy and diseased RVs. Red represents significantly upregulated APA factors, while blue represents significantly downregulated APA factors. Unchanged genes are depicted in grey. p-adjusted values less than 0.05 and log2-fold change of more than or equal to 1, or less than or equal to − 1, respectively.

The length of the 3’UTR significantly impacts mRNA fate, thereby influencing its stability [51]. To dissect this further, we assessed the differential expression of these genes to identify differentially expressed genes (DEG) in healthy vs. diseased RVs. Decompensated RVs displayed 8,631 DEGs compared to control RVs, of which 4,320 were upregulated and 3,412 were downregulated (Figure 3A). Similarly, decompensated RVs exhibited 5,263 DEGs relative to compensated RVs, with 2,648 upregulated and 2,270 downregulated (Figure 3B). There were also a notable number of DEGs in compensated RVs compared to control RVs, though far fewer than in decompensated RVs, with 408 genes upregulated and 291 genes downregulated (Figure 3C). We also analyzed the biological pathways associated with the DEGs to gain deeper insight into the mechanisms driving decompensation and RV failure. In the control vs. decompensated RV comparison, the upregulated DEGs were enriched for pathways related to stress-responsive signaling, growth and developmental processes, vascular remodeling, cytoskeletal organization, and inflammatory responses, consistent with an active but maladaptive attempt to preserve cardiac output through hypertrophic and remodeling mechanisms (Supplementary Figure S2A). In contrast, the downregulated DEGs were predominantly associated with mitochondrial and metabolic energy pathways, including fatty acid oxidation, oxidative metabolism, protein turnover, autophagy, and intracellular transport, indicating impaired energetic capacity and disrupted cellular homeostasis in the decompensated RV (Supplementary Figure S2B). In the compensated vs. decompensated RV comparison, the upregulated DEGs were primarily enriched for adaptive signaling pathways, developmental and morphogenetic programs, cell migration, angiogenesis, and vasculature development, reflecting the relative preservation of compensatory remodeling responses in compensated RVs (Supplementary Figure S2C). Conversely, the downregulated DEGs were strongly associated with core metabolic and bioenergetic processes, as well as cardiac contractile, electrophysiological, and conduction pathways, suggesting progressive loss of cardiomyocyte functional integrity and energetic efficiency during RV decompensation (Supplementary Figure S2D). A complete list of enrichment analyses is provided in Supplementary Table S2. Together, these findings indicate that RV decompensation is characterized by activation of stress and remodeling pathways alongside a broad suppression of metabolic, mitochondrial, and cardiomyocyte functional programs, distinguishing maladaptive failure from compensated remodeling.

### APA Dysregulation Is Recapitulated in Rat Models of PAH-Associated RV Failure

**FIGURE 4:**
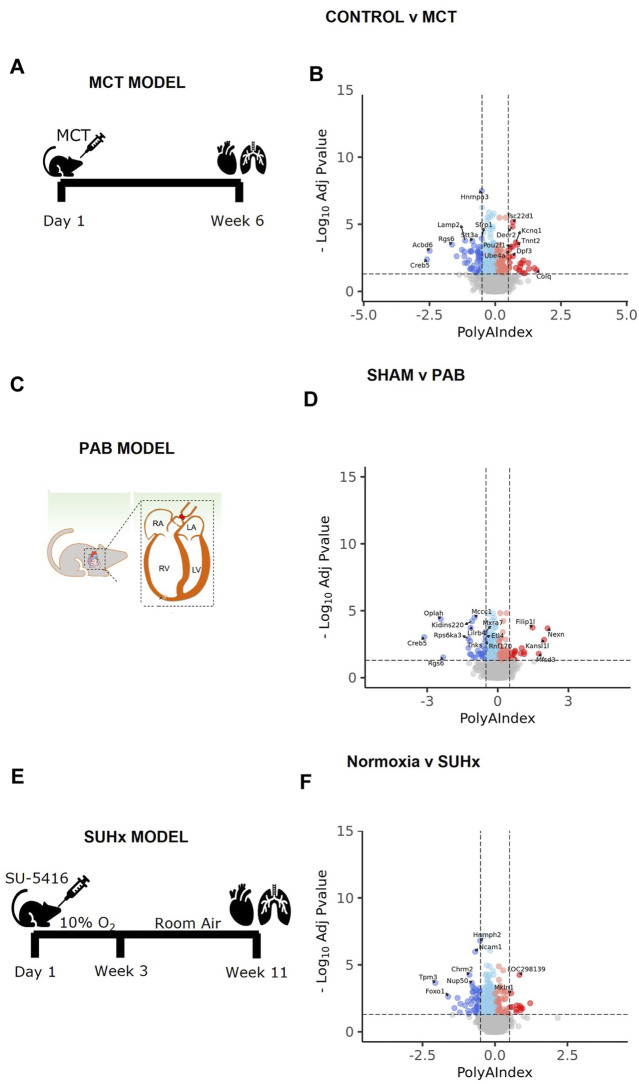
Several rat models of PH-related RV failure recapitulate APA dysregulation events. Schematic representation of rat model PH-associated RV failure in **(A)** MCT, **(B)** PAB, and **(C)** SUHx rat models. Volcano plots illustrate transcript length alterations in **(D)** Control vs. MCT, **(E)** Sham vs. PAB, and **(F)** Normoxis vs. SUHx rat models, where dark red = significantly elongated (SigEl), light red = elongated (El), dark blue = significantly shortened (SigSh), light blue = shortened (Sh) and grey = unchanged (NoSig) transcripts. The horizontal dashed lines indicate the −log_10_ (*P* adjusted) ≥1.325 (*P* adjusted ≤0.05), and the vertical dashed lines indicate polyA index ≥+0.1 and ≤−0.1, with positive values indicating longer transcripts and negative values indicating shorter transcripts.

To further investigate the mechanisms underlying RV failure and establish a solid platform for developing and testing therapeutic interventions, we utilized three animal models of PH-associated RV failure: the MCT, the PAB and the SuHx models of PH. The MCT model involves administering monocrotaline, a toxic pyrrolizidine alkaloid, which induces pulmonary vascular injury and subsequent PH (Figure 4A). This model is popular due to its simplicity and ability to cause severe PH and RV failure [52]. DAG analysis revealed 101 elongated transcripts and 257 transcripts with shorter 3’UTRs (Figure 4B). The PAB model surgically constricts the pulmonary artery, increasing RV afterload without affecting the pulmonary vasculature, enabling researchers to isolate the effects of increased afterload on the RV. It also allows for variable levels of constriction or loosening of the PA, providing insight into afterload effects on RV remodeling (Figure 4C) [52]. Using this model, we reveal 74 transcripts with elongated 3’UTRs and 162 transcripts with shorter 3’UTRs (Figure 4D). The SuHx model combines a vascular endothelial growth factor receptor (VEGFR) inhibitor, Sugen 5416, with chronic hypoxia, closely mimicking the progressive and severe nature of human PAH, resulting in significant pulmonary vascular remodeling and severe RV failure (Figure 4E) [52]. In this model, we identified 76 transcripts with longer 3’UTRs and 234 transcripts with shorter 3’UTRs (Figure 4F). A complete list of APA analyses is provided in Supplementary Table S3.

Subsequent pathway analysis revealed parallels with human decompensated RVs. DAGs in both cases were significantly associated with pathways, including the longevity regulating pathway, Mitogen-activated protein kinases (MAPK) signaling, and adrenergic signaling in cardiomyocytes and ECM organization (Supplementary Figure S3). However, some key differences revealed how, in the MCT model, DAGs were implicated in pathways such as ubiquitin-mediated proteolysis and cardiomyopathy (Supplementary Figure S3A). In the PAB model, DAGs mapped to pathways such as neurotrophin signaling and ErbB (receptor tyrosine kinase) signaling (Supplementary Figure S3B). As seen in the MCT model, there were notable overlaps with human RV failure, including involvement in insulin signaling and MAPK signaling pathways. In the SUHx model, with significant DAGs enriched in pathways such as protein processing in the endoplasmic reticulum and the Citrate cycle (TCA cycle) (Supplementary Figure S3C). In line with aspects of human RV failure, these DAGs also influenced pathways like insulin signaling and cardiomyopathy. Interestingly, while similar pathways were altered, the general trend of altered transcripts was flipped, where the human disease had more transcripts with lengthened 3’UTRs. Yet, the rat models demonstrated more transcripts with shortened 3’UTRs.

**FIGURE 5:**
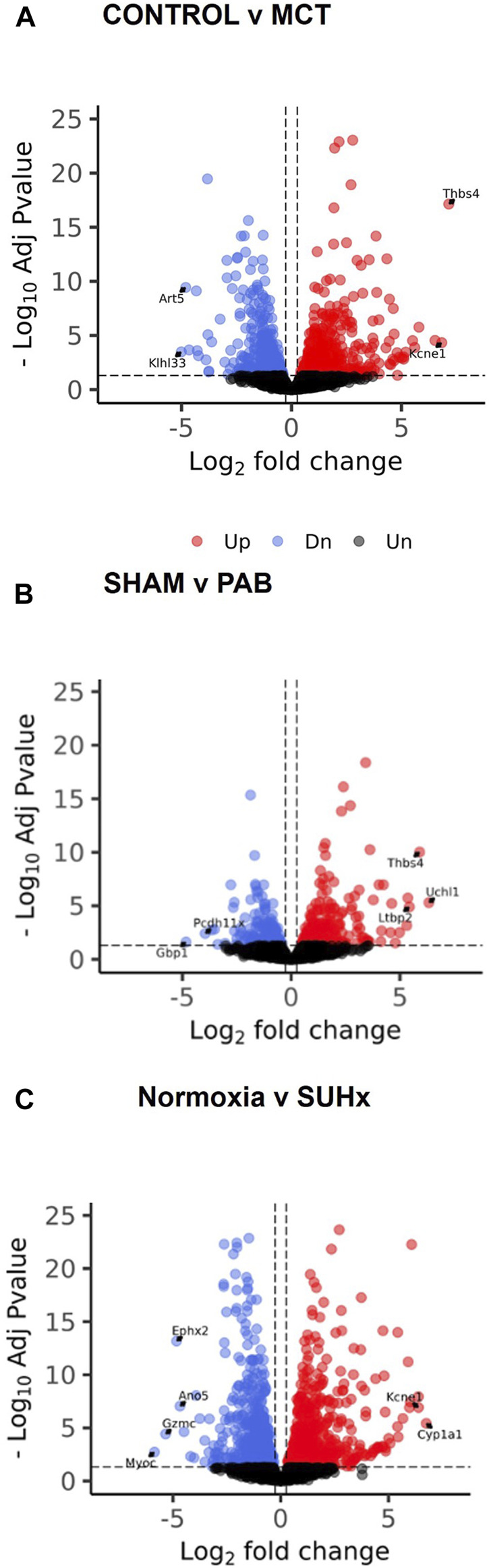
Transcriptomic analysis of 3 PH-associated RV failure rat models shows broad gene expression dysregulation. Volcano plots identify DEGs in **(A)** MCT, **(B)** PAB, and **(C)** SUHx models of RV failure. Red represents significantly upregulated APA factors, while blue represents significantly downregulated APA factors. Unchanged genes are depicted in grey. p-adjusted values less than 0.05 and log2-fold change of more than or equal to 0.263, or less than or equal to – 0.263, respectively.

Significant differential gene expression was observed across the three rat models of PH-associated RV failure. In the MCT model, 1019 DEGs were identified, with 551 genes upregulated and 468 downregulated (Figure 5A). The PAB model displayed 482 DEGs, consisting of 276 upregulated and 206 downregulated genes (Figure 5B). The SUHx model exhibited the highest number of DEGs, with 3570 total DEGs, including 1817 upregulated and 1753 downregulated genes (Figure 5C).

Pathway enrichment analysis of the DEGs revealed significant similarities between the three rat models and human RV failure. In all models, upregulated genes were consistently associated with pathways involved in Extracellular Matrix (ECM) Organization and inflammation, reflecting the pathological remodeling processes observed in human disease (Supplementary Figures S4–S6). These changes highlight the shared mechanisms of structural remodeling and immune response activation in both experimental and clinical RV failure. Conversely, downregulated genes were predominantly linked to pathways involving metabolism and mitochondrial respiration, suggesting a disruption in energy production and metabolic homeostasis, which are critical for maintaining cardiac function under stress (Supplementary Figures S4–S6).

These findings emphasize the utility of the three rat models in recapitulating key molecular aspects of human RV failure. Additionally, the convergence of molecular pathways across both species provides critical insights into the pathological mechanisms of RV failure, supporting the use of these models for future therapeutic exploration.

### Utilizing APA to Identify Key Molecular Targets and Pathways Driving RV Dysfunction

**FIGURE 6:**
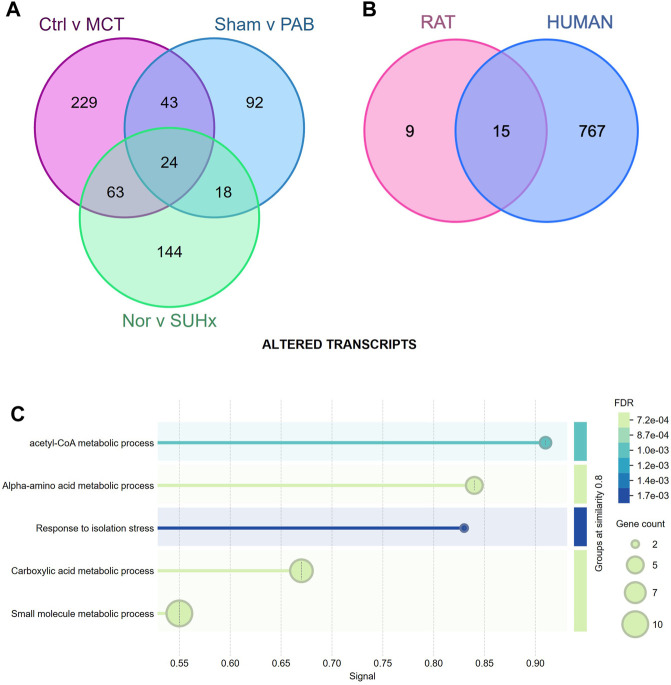
Common and distinct APA changes drive disease pathology in RV failure and rat models. Venn diagrams cluster common APA events, producing transcripts of altered lengths in **(A)** three rat models, and **(B)** across the rat models and human disease. Of all APA events noted, 15 genes show dysregulated APA in all 3 rat models as well as human disease, pinpointing some common drivers of RV pathology. **(C)** Pathway enrichment analysis of the 15 common genes driving disease isolate mechanisms that may drive RV pathology.

APA alters the length of mRNA, influencing their stability, localization, and translation, affecting gene expression profiles during RV decompensation. Comparative analysis of rat models of RV failure (Figure 6A) and human decompensated RVs revealed common APA changes (Figure 6B), pointing to conserved mechanisms underlying disease progression. Notably, we identified 15 genes with APA dysregulation common to both rat models and human RV decompensation (Table 1). The 15 genes—*AGPAT3, ALDH5A1, ANGEL2, AZIN1, DLAT, DNAJA2, EPB41L1, FDFT1, MAPK9, MXRA7, NAMPT, SERBP1, SOD2, SPRYD7, and STXBP5*—play crucial roles in regulating metabolism and stress responses in RV failure (Figure 6C). These genes are involved in lipid and energy metabolism, protein folding, and antioxidant defense and could collectively lead to exacerbating disease progression. For example, *AGPAT3* and *FDFT1* affect lipid profiles and membrane integrity [53, 54], *DLAT* and *NAMPT* are key players in energy production [55, 56], *DNAJA2* and *SOD2* provide protective mechanisms against oxidative stress [57, 58], and *MAPK9* mediates inflammatory and hypertrophic responses [59]. Dysregulation of these genes by APA can impair the RV’s ability to adapt to stress, leading to metabolic disturbances and promoting dysfunction. Therefore, these insights not only enhance our understanding of the disease but also present promising molecular targets for therapeutic intervention while also offering new opportunities to modulate disease progression at the post-transcriptional level.

**TABLE 1:** Patient demographics.

Sample ID	Sample Type	Donor age	Sex	Cause of death/Diagnosis	Use
CLT RV 1-1	Control RV	52	F	Sudden death	WB1
CLT RV 1-2	Control RV	65	M	Sudden death	WB1
CLT RV 1-3	Control RV	49	F	Sudden death	WB1
CLT RV 1-4	Control RV	29	M	Aortic valve stenosis	WB1
cRV 1-1	Compensated RV	29	M	Pulmonary valve regurgitation	WB1
cRV 1-2	Compensated RV	27	M	Pulmonary valve stenosis	WB1
cRV 1-3	Compensated RV	54	F	Pulmonary valve stenosis	WB1
cRV 1-4^a^	Compensated RV	18	F	Pulmonary valve stenosis	WB1
dRV1-1	Decompensated RV	47	F	Porto pulmonary hypertension	WB1
dRV1-2	Decompensated RV	77	F	SSc-PAH	WB1
dRV1-3	Decompensated RV	72	F	PAH capillary pulmonary hemagiomatiosis with VOD	WB1
CLT RV 2-1	Control RV	53	M	Aortic valve stenosis	WB2
CLT RV 2-2	Control RV	56	M	Aortic valve stenosis	WB2
CLT RV 2-3	Control RV	46	F	Aortic regurgitation	WB2
cRV 2-1	Compensated RV	30	F	Idipathic RV dilation	WB2
cRV 2-2	Compensated RV	61	F	Ventricular septal defect	WB2
cRV 2-3	Compensated RV	32	F	Pulmonary valve stenosis	WB2
dRV2-1	Decompensated RV	54	F	SSc-PAH	WB2
dRV2-2	Decompensated RV	77	M	SSc-PAH	WB2
dRV2-3	Decompensated RV	47	F	SSc-PAH	WB2
dRV2-4	Decompensated RV	75	F	IPAH	WB2
dRV2-5	Decompensated RV	57	F	CHD-PAH	WB2
CTL RV 3-1	Control RV	47	M	Aortic valve stenosis	WB3
CTL RV 3-2	Control RV	22	M	Aortic valve stenosis	WB3
CTL RV 3-3	Control RV	58	M	Aortic valve stenosis	WB3
CTL RV 3-4	Control RV	55	M	Aortic valve stenosis	WB3
cRV 3-1	Compensated RV	28	F	Tricuspid valve defect	WB3
cRV 3-2	Compensated RV	23	F	Pulmonary valve regurgitation	WB3
cRV 3-3	Compensated RV	23	F	Ebstein anomaly	WB3
dRV3-1	Decompensated RV	53	F	SSc-PAH	WB3
dRV3-2	Decompensated RV	61	F	HPAH	WB3
dRV3-3	Decompensated RV	65	M	IPAH	WB3
dRV3-4	Decompensated RV	72	F	PVOD	WB3

^a^ Not available for NUDT21 WB.

### Global Dysregulation of APA Factors and Mediators in RV Dysfunction

Our data demonstrates significant changes in the 3’UTR landscape in RV dysfunction in both patients and in experimental models of disease. Given that alternative polyadenylation is orchestrated by the cleavage and polyadenylation (CPA) machinery, we examined whether select components of this complex are implicated in the observed APA dysregulation. Previous studies have demonstrated the role of CPA complex proteins in regulating APA. For example, knockdown of *NUDT21* leads to global 3' UTR shortening and promotes disease-associated gene expression changes [32, 39]. Consistent with this framework, we observed altered abundance of the CPA component CPSF5 at the protein level in RV dysfunction (Figure 2), providing orthogonal evidence that dysregulation of the polyadenylation machinery accompanies APA remodeling in disease. Together, these findings support the notion that APA changes reflect underlying perturbations in CPA-mediated RNA processing and highlight APA as a reliable indicator of RV dysfunction.

**FIGURE 7:**
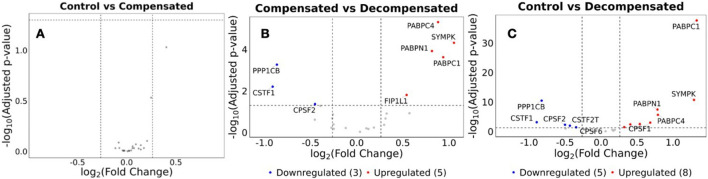
Multiple APA mediators are differentially expressed in compensated and decompensated RVs. Differential gene expression of APA factors and mediators from **(A)** Control vs. Decompensated, **(B)** Compensated vs. Decompensated, and **(C)** Control vs. Compensated RVs. Red genes represent significantly upregulated APA factors, while blue represents significantly downregulated APA factors. Unchanged genes are depicted in grey. p-adjusted values less than 0.05 and log2-fold change of more than or equal to 1, or less than or equal to – 1, respectively.

Given the widespread APA dysregulation observed in RV decompensation, we investigated if the core cleavage and polyadenylation (CPA) machinery presented with altered gene expression. Supporting the dysregulated APA, we found that several components of the CPA complex are differentially expressed in decompensated RVs (Figures 7B,C). Eight CPA complex components are upregulated and five are downregulated when comparing decompensated RVs with control RVs (Figure 7C). Among the most prominent changes, *PABPC1*, *SYMPK, PABPN1, PABPC4,* and *CPSF1* are upregulated in decompensated RVs, whereas *PPP1CB, CSTF1, CPSF2, CPSF6* and CSTF2T are reduced. By comparison, analysis of compensated vs. decompensated RVs reveals 5 upregulated and 3 downregulated CPA components (Figure 7B). The upregulated genes include *PABPC1*, *PABPC4*, *PABPN1*, *SYMPK*, and *FIP1L1*, whereas *PPP1CB, CSTF1,* and *CPSF2* are downregulated. In line with the minimal dysregulation of APA observed in compensated RVs, we detected no significant changes to the CPA complex in control vs. compensated groups (Figure 7A). Further analysis of DEGs of the CPA complex components shows that *CPSF6*, and *CSTF2T* are significantly downregulated in the decompensated RVs. At the same time, *CPSF1, RBBP6 and PCF11* are significantly upregulated in the decompensated RV, but not in compensated or control RV. Our data shows that APA serves as a reliable indicator for understanding RV dysfunction.

### Validation of APA Targets

**FIGURE 8:**
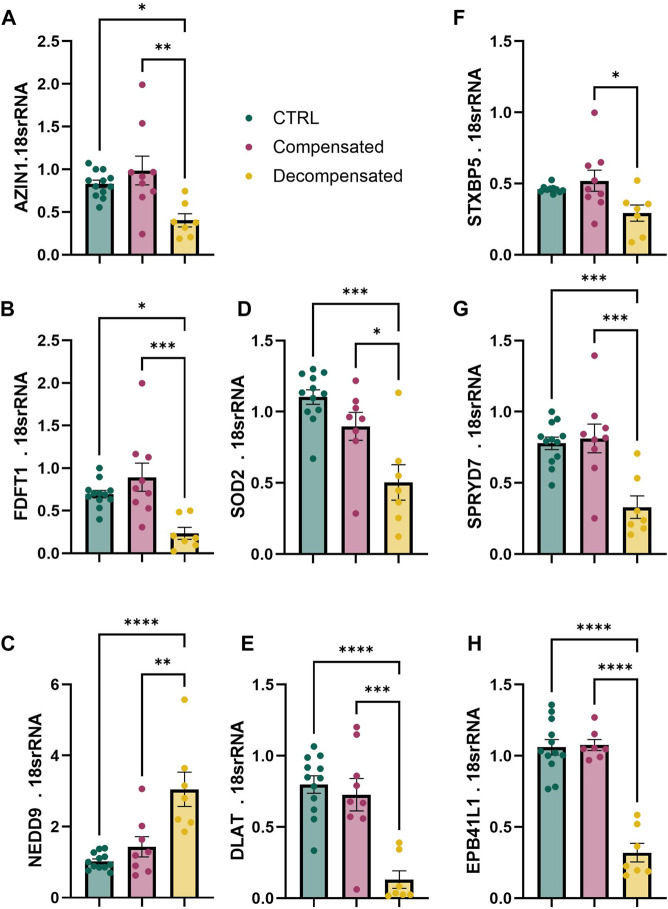
Gene expression from transcripts presenting with APA in human right ventricle (RV) tissue. mRNA expression levels using RT-qPCR for *AZIN1* **(A)**, *FDFT1* **(B)**, *NEDD9* **(C)**, *SOD2* **(D)**, *DLAT* **(E)**, *STXBP5* **(F)**, *SPRYD7* **(G)**, and *EPB41L1* **(H)**, from control (N = 12 green), compensated (N = 9, red) and decompensated (N = 7, yellow) human right RV tissue. Significance levels*p˂0.05, ***p˂0.01, and ***p˂0.001 refer to comparisons between control or compensated RV vs. decompensated RV.

**FIGURE 9:**
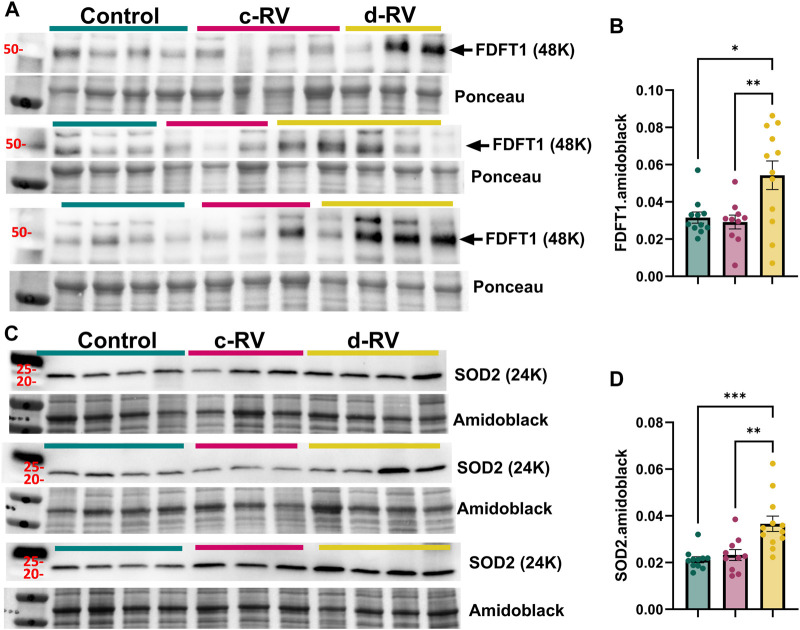
Increased expression of farnesyl-diphosphate farnesyltransferase 1 (FDFT1) and superoxide dismutase 2 (SOD2). Western blots for FDFT1 and control ponceau stain **(A)** and subsequent quantification **(B)**. Immunoblot for SOD2 and control Amidoblack **(C)** and subsequent quantification **(D)**. Experiments were performed from isolated control (N = 11, teal), compensated (N = 9, pink) and decompensated RVs (N = 12, yellow). Significance levels*p˂0.05, ***p˂0.01, and ***p˂0.001 refer to comparisons between control or compensated RV vs. decompensated RV.

**FIGURE 10:**
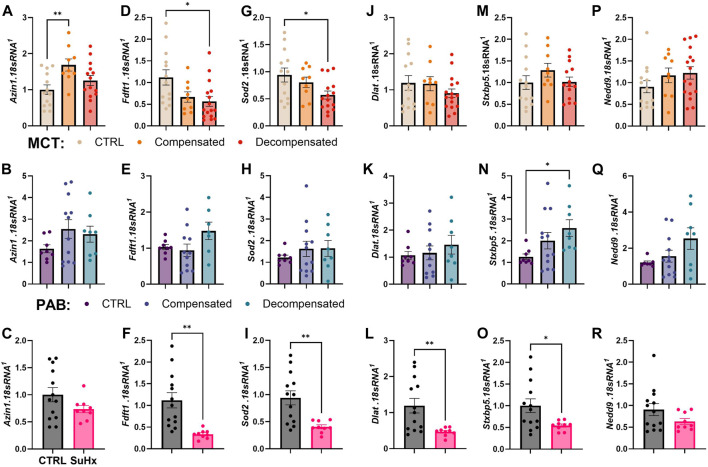
Gene expression of transcripts presenting with APA in mouse models presenting right ventricle (RV) dysfunction. mRNA expression levels using RT-qPCR for *Azin1* **(A–C)**, *Fdft1* **(D–F)**, *Sod2* **(G–I)**, *Dlat* **(J–L)**, *Stxbp5* **(M–O)**, and *Nedd9* **(P–R)**, from RVs from the monocrotaline (MCT) rat model control (N = 13 yellow), compensated (N = 9, orange) and decompensated (N = 17, red) RVs from; the pulmonary artery banding (PAB) model: control (N = 8 purple), compensated (N = 12, dark blue) and decompensated (N = 8, light blue) and from the sugen hypoxia (SuHx) rat model: control (N = 13, grey) or SuHx exposure (N = 9, pink). Significance levels*p˂0.05, ***p˂0.01, and ***p˂0.001 refer to comparisons between control or compensated RV vs. decompensated RV.

Next, we validated gene and protein levels of differential APA targets identified in by qPCR and Western blot in both human and rat RVs. We utilized control RV (n = 11), compensated RV (n = 11) and decompensated RV (n = 12) tissues from the Biorepository of Centre de recherche de l'Institut universitaire de cardiologie et de pneumologie de Québec; these represent distinct samples from the RVs used for bioinformatic analyses. Herein, we identified reduced expression of *AZIN1, FDFT1, SOD2, DLAT, SPRYD7,* and *EPB41L1* in decompensated RV compared to control or compensated RV groups (Figures 8A–F). *STXBP5* expression was also reduced in decompensated TV compared to compensated RV (Figure 8G). However, *NEDD9* expression levels were elevated in decompensated RVs compared to both control and decompensated RVs (Figure 8H). From the 15 conserved APA targets, we prioritized *FDFT1* and *SOD2* for protein validation because they map to two core RV decompensation hallmarks: lipid/sterol metabolism and mitochondrial oxidative stress, while also being highly plausible points of APA-driven post-transcriptional control. Herein, we report increased expression of these mediators in decompensated RVs compared to control or compensated RVs (Figure 9A,B). We also determined expression of these targets in our rat models of RV dysfunction (Figure 10). Herein, *Azin1* was upregulated only in compensated RVs in MCT model with no changes in either the PAB or SuHx models (Figures 10A–C). *Fdft1* and *Sod2* expression were both reduced in decompensated RVs in the MCT and in the SUHX models, with no changes seen in the PAB model (Figures 10D–I). Dlat expression levels were only reduced in the SUHx-treated mice with no changes in either MCT or PAB models (Figures 10J–L). Stxb5 expression levels were unaltered in the MCT model, elevated in the decompensated RV from the PAB model, and reduced in SUHx-treated rats (Figures 10M–O). No changes in *Nedd9* expression were reported in any experimental model (Figures 10P–R). Taken together, these results demonstrate differences in gene expression between experimental models of RV dysfunction.

## Discussion

This study demonstrates that APA is a defining molecular feature of decompensated RV failure. By integrating transcriptomic profiling and APA analysis of human RV tissue, we show that global 3′UTR elongation is specifically associated with decompensated RV remodeling, while compensated RVs exhibit minimal APA disruption. The distinct enrichment of lengthened 3′UTRs in decompensated RVs could serve as a valuable tool for risk stratification and for identifying patients at higher risk of progression. These findings highlight APA signatures as potential biomarkers capable of distinguishing adaptive from maladaptive RV responses in PAH.

Prior work has established that 3′UTR shortening frequently accompanies proliferative and fibrotic conditions [36, 37, 39], while elongation is linked to differentiation, senescence, and cellular stress [40–42]. In our study, extensive 3′UTR lengthening in failing RVs suggests maladaptive attempts to regulate mRNA stability and translation under chronic pressure overload. Enrichment analyses revealed involvement of stress response and metabolic pathways, including AMPK, p53, HIF-1A, and insulin signaling [60–62], which are crucial for managing cellular stress, metabolic regulation, and hypoxic responses [60–62].

Notably, our three animal models (MCT, PAB, and SuHx) recapitulated many transcriptomic changes observed in human disease, including alterations in pathways regulating extracellular matrix remodeling, inflammation, and metabolism. Despite these similarities, APA dynamics diverged: the rat models exhibited a trend toward 3′UTR shortening. This discrepancy may reflect differences in disease chronicity, as RV remodeling in experimental models evolves over weeks rather than years [63]. Because elongated transcripts often incorporate additional regulatory elements that influence mRNA stability and translation, 3′UTR lengthening in the failing human heart could represent a compensatory mechanism to modulate protein expression under chronic stress. Alternatively, the divergence may result from differences in disease stage. While SuHx rats develop and sustain PH, RV remodeling and dysfunction typically improve when animals are returned to normoxia [63], suggesting the transcriptomic profile may more closely reflect a compensated state. Nevertheless, the convergence of affected pathways highlights the value of these models for investigating APA regulation and evaluating targeted interventions.

Among the most significant findings was the identification of 15 transcripts showing conserved APA dysregulation across species. These genes are central to lipid metabolism (*AGPAT3, FDFT1*), oxidative stress defense (*SOD2*), energy production (*DLAT, NAMPT*), and inflammatory signaling (*MAPK9*), studied in the context of metabolic disorders, seizure disorders, and cancer therapy, [53–55, 57–59]. This implies that APA dysregulation could compromise the ability of cells to adapt and survive under the metabolic stress and hypoxia characteristic of PAH. Several of these targets have been studied in other cardiovascular or metabolic contexts, including the modulation of *SOD2* with antioxidants [64] or indirect inhibition of *FDFT1* through statins [65]. The observed discrepancies between transcript and protein levels of *SOD2* and *FDFT1* further illustrate the complexity of post-transcriptional regulation, suggesting that APA impacts not only mRNA abundance but also translational control and protein turnover [29].

Our findings further suggest that perturbation of the cleavage and polyadenylation (CPA) machinery accompanies APA remodeling in decompensated RVs. Prior studies have shown that key CPA factors, including NUDT21 and the protein it encodes, CPSF5, play central roles in shaping the 3′UTR landscape. Consistent with this framework, we observed altered abundance of CPSF5 at the protein level in decompensated RVs, supporting a mechanistic link between CPA machinery disruption and widespread 3′UTR elongation. Further analysis of differentially expressed genes (DEGs) within the CPA complex revealed that *CPSF6* and *CSTF2T* are significantly downregulated in decompensated right ventricles (RVs). In contrast, *CPSF1*, *RBBP6*, and *PCF11* are significantly upregulated specifically in decompensated RVs, with no significant changes observed in compensated or control RVs. Together, these observations implicate dysregulated RNA processing rather than global transcriptional changes as a contributor to maladaptive RV remodeling.

In contrast, compensated RVs exhibited minimal APA disruption, reinforcing the idea that widespread APA remodeling emerges as a maladaptive feature during the transition to RV failure. The absence of pronounced CPA-associated APA changes in the rat models of PH-associated RV failure was unexpected, given the presence of APA dysregulation in these animals. This discrepancy may reflect differences in disease chronicity, as experimental RV remodeling progresses over weeks, whereas human RV failure evolves over years. Similar to the reversal in 3′UTR length patterns, the CPA gene expression profiles in rat RVs likely represent an intermediate state along the continuum from compensation to decompensation.

These results have important clinical implications. The identification of disease-specific APA patterns and CPA complex dysregulation provides a foundation for developing RNA-based biomarkers to monitor RV remodeling and stratify patients by risk of decompensation. In line with this, our findings are consistent with pathway-level alterations previously reported by Khasaffi et al. [21], with alterations in immune pathways, ECM regulation, PI3-Akt signaling, HIF-1A signaling, metabolism, cytoskeletal organization, and mitochondrial function. Furthermore, targeting APA regulators may offer novel therapeutic opportunities to modify maladaptive gene expression programs in RV failure. Given the lack of effective RV-specific therapies in PAH [3], such strategies could address a major unmet need.

Limitations of this study should be acknowledged. Our analyses relied on bulk RV tissue, potentially masking cell type–specific APA events. While we validated key transcripts at the mRNA and protein levels, causal relationships between APA changes and RV dysfunction remain to be established through functional studies. Additionally, sample sizes for human tissue were modest, reflecting the rarity of well-characterized RV specimens. Finally, the use of rat models, while informative, may not fully recapitulate the chronicity and complexity of human disease.

In conclusion, our study identifies APA dysregulation as a defining molecular feature of decompensated RV failure. By positioning APA as both a diagnostic biomarker and a potential therapeutic target, this work bridges fundamental RNA biology with translational applications, aligning with the growing field of RNA-based diagnostics and therapeutics.

## Data Availability

The datasets presented in this study can be found in online repositories. The names of the repository/repositories and accession number(s) can be found below: Gene Expression Omnibus (GEO) database (accession number: GSE198618).
