# Supplementary material for: Alternative Polyadenylation Signatures Distinguish Maladaptive Right Ventricular Remodeling in Pulmonary Hypertension: Implications for RNA-Based Diagnostics and Therapeutics
Source: Br J Biomed Sci. 2026 Feb 20;83:15687. doi: 10.3389/bjbs.2026.15687 (PMC12963017; doi:10.3389/bjbs.2026.15687)
Supplement: Supplementary file 3 [file DataSheet1.pdf]

SUPPLEMENTARY FIGURE 1

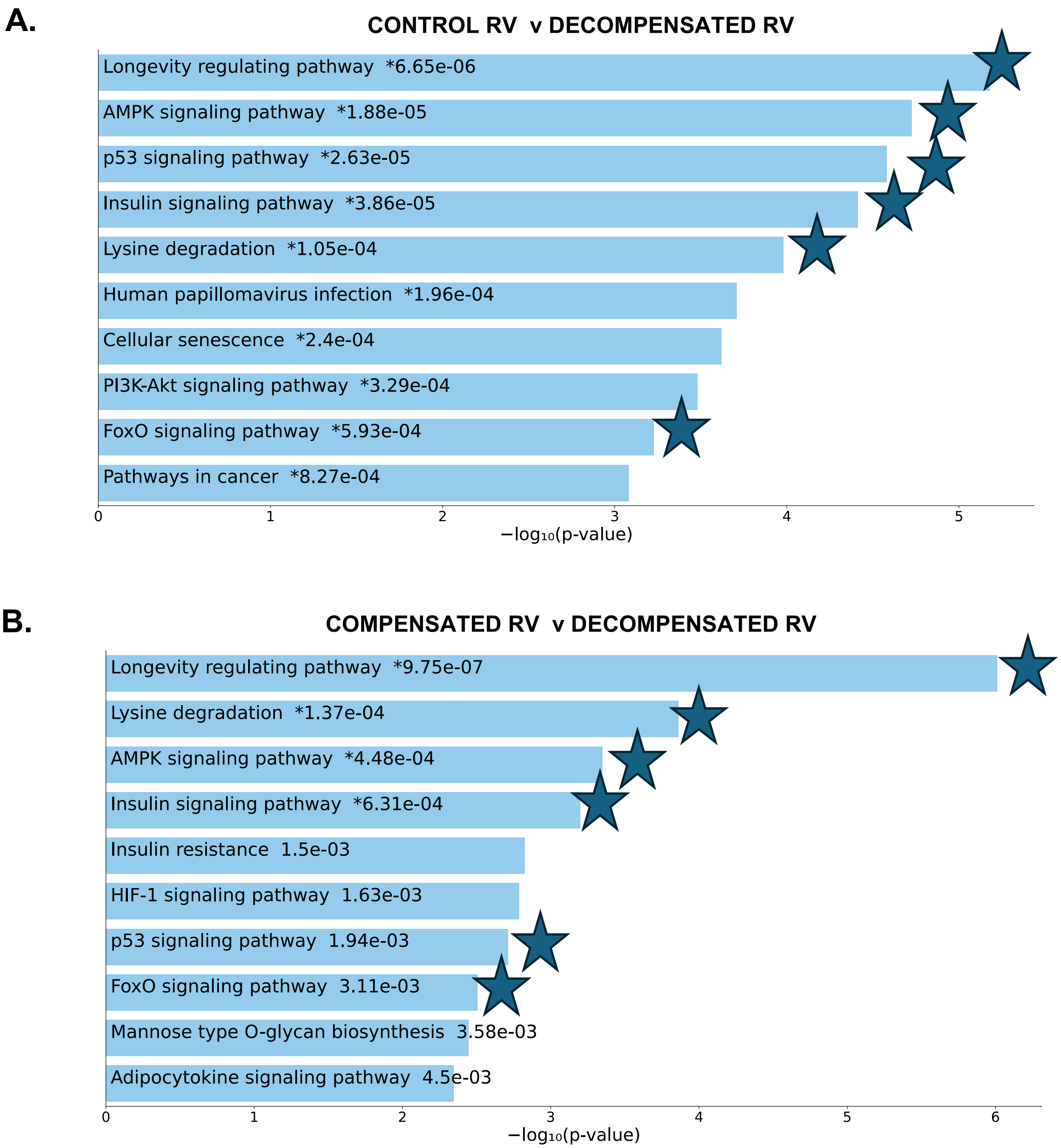

Supplementary Figure 1. Common Enriched Pathways arising from alternative polyadenylation (APA) in Decompensated Human RVs. Top highlighted differentially enriched pathways driven by altered transcript length, with associated p-value in each comparison, **(A)** Control vs. Decompensated, and **(B)** Compensated vs. Decompensated RVs. Blue stars denote common pathways between both analyses.

SUPPLEMENTARY FIGURE 2

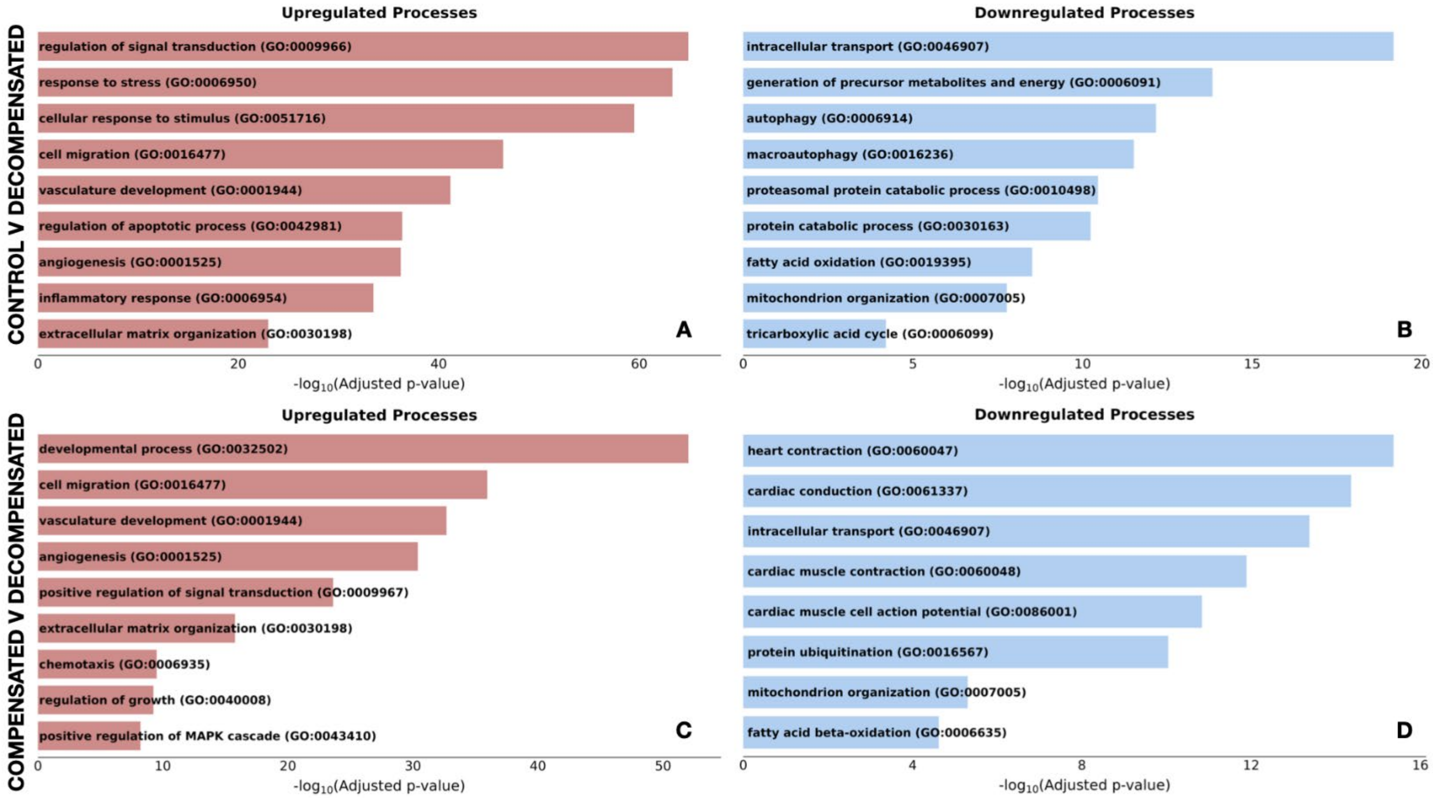

Supplementary Figure 2. Altered pathways in the decompensated RV. Pathways analysis of DEGs uncover several upregulated (red) and down regulated (blue) pathways that could contribute toward disease progression of decompensated RV failure in each comparison, **(A, B)** Control vs. Decompensated, and **(C, D)** Compensated vs. Decompensated RVs. DEGs= Differentially expressed genes.

SUPPLEMENTARY FIGURE 3

A. CONTROL v MCT

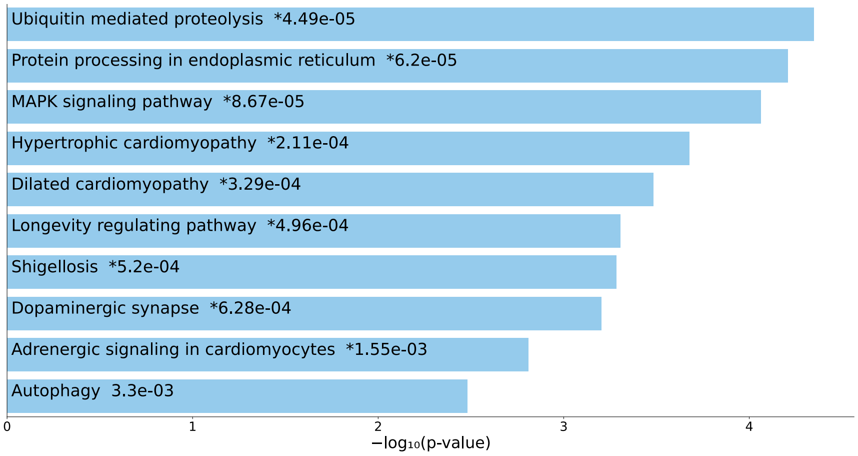

B. SHAM v PAB

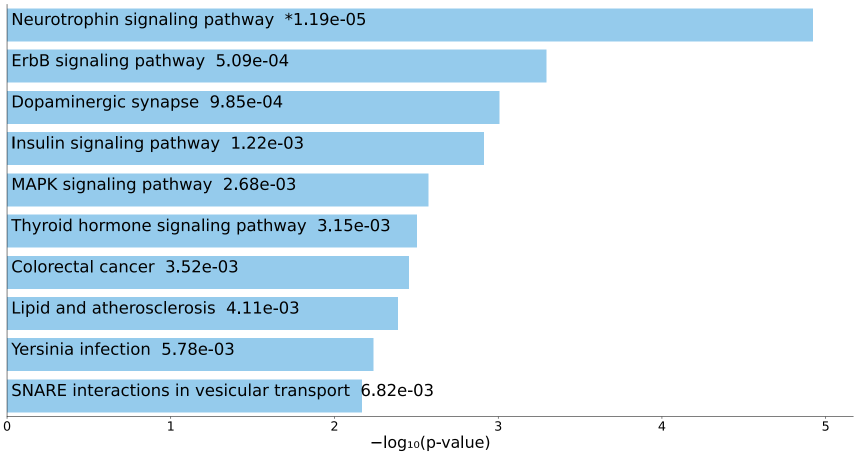

C. NORMOXIA v SUHx

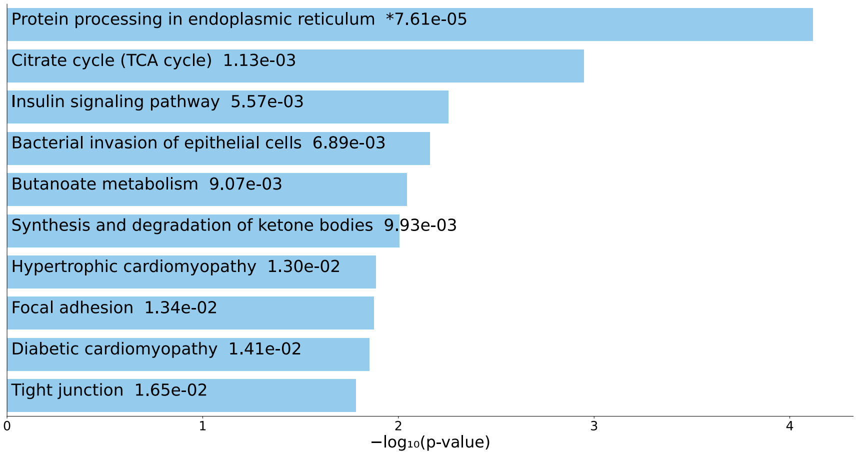

Supplementary Figure 3. Altered pathways arising from alternative polyadenylation (APA) in rat models of RV dysfunction. Top highlighted differentially enriched pathways driven by altered transcript length, with associated p-value in each comparison, in **(A)** MCT, **(B)** PAB, and (**C)** SUHx rat models. MCT= Monocrotaline, PAB = Pulmonary Arterial Banding, SUHx = Hypoxia/Sugen

SUPPLEMENTARY FIGURE 4

CONTROL v MCT

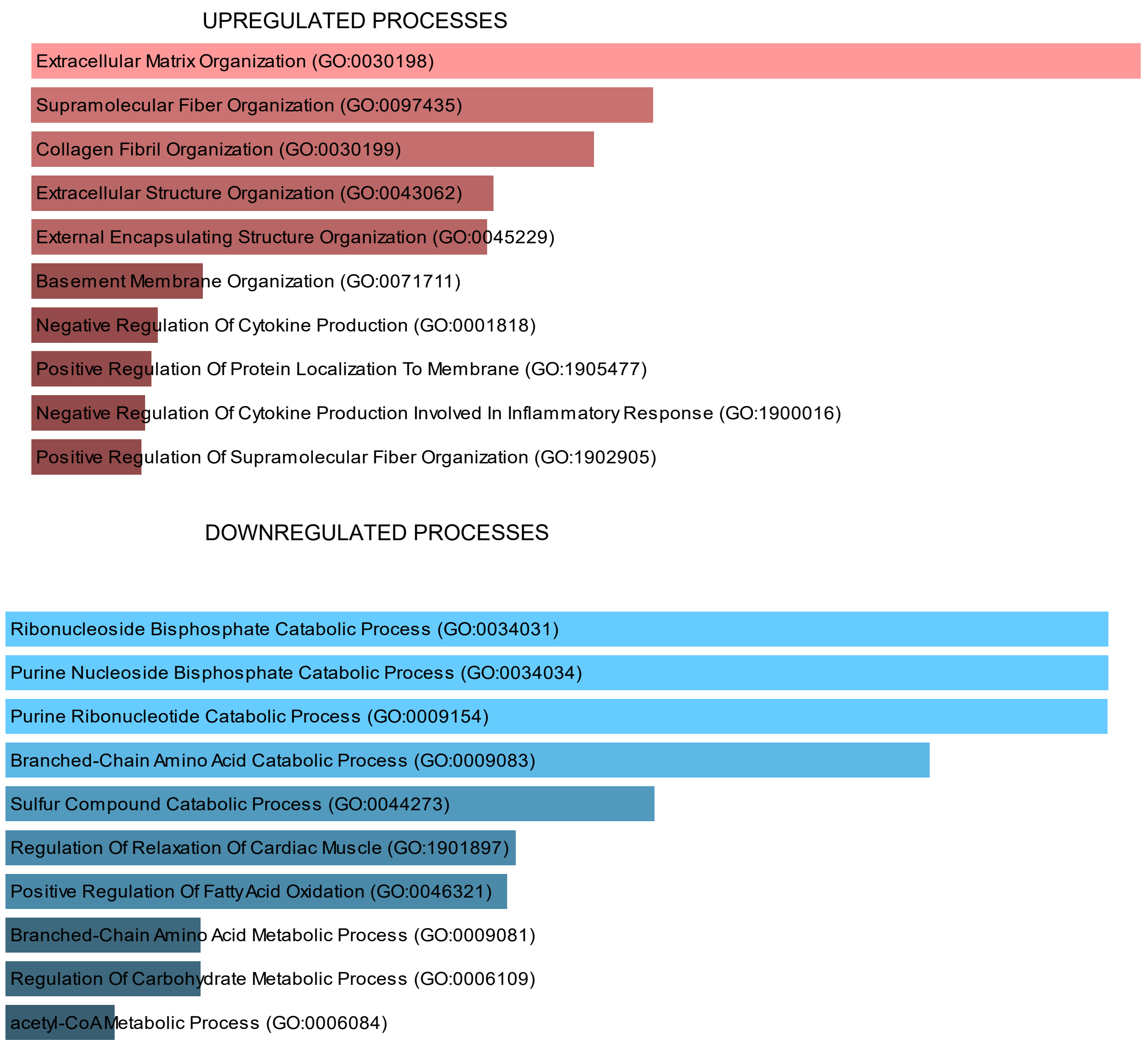

Supplementary Figure 4. Pathway enrichment analysis of DEGs reveal distinctly upregulated (red) and downregulated (blue) pathways that could contribute to RV decompensation in the rat monocrotaline (MCT) model

SUPPLEMENTARY FIGURE 5

SHAM v PAB

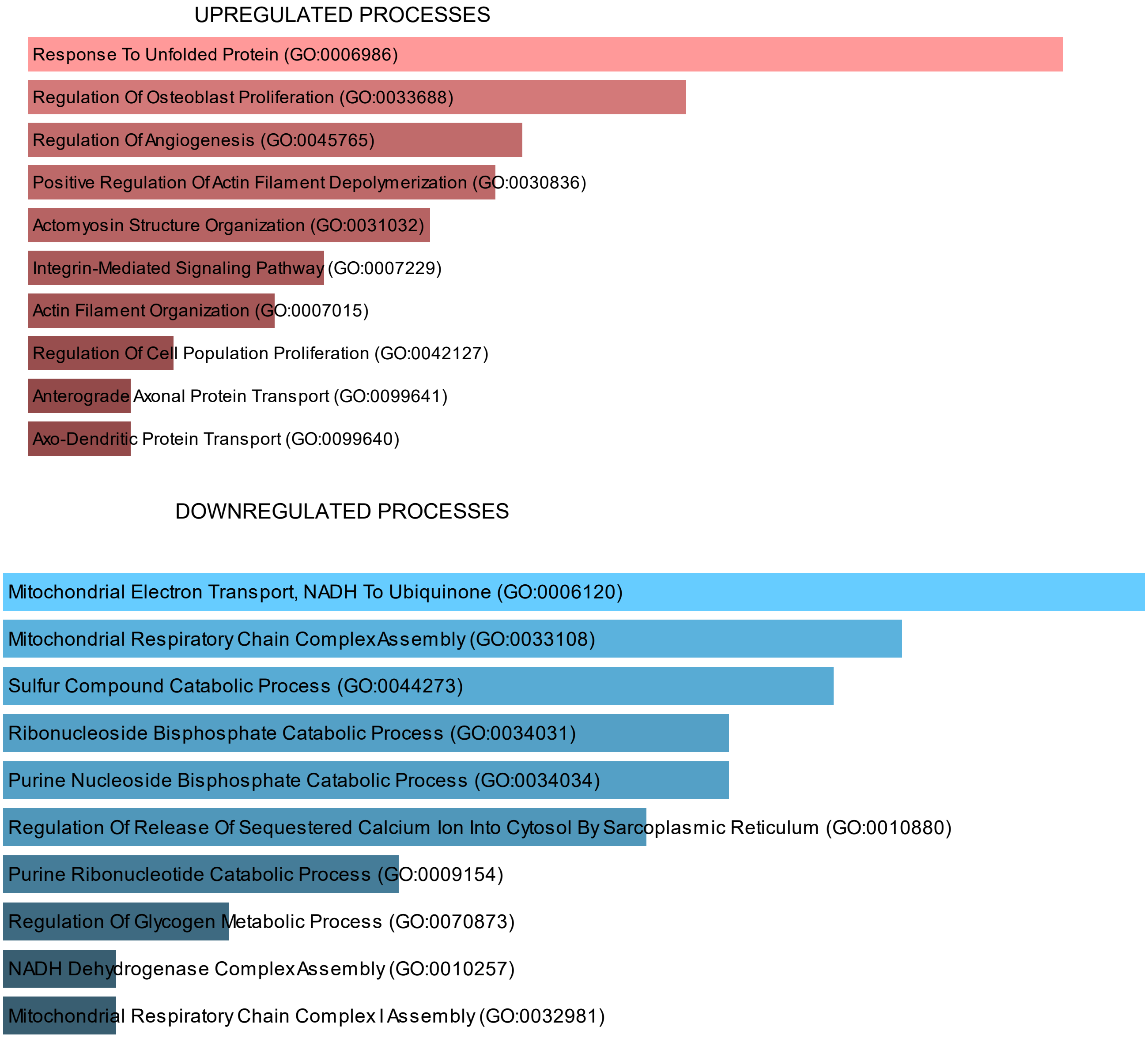

Supplementary Figure 5. Pathway enrichment analysis of DEGs reveal distinctly upregulated (red) and downregulated (blue) pathways that could contribute to RV decompensation in the pulmonary artery banding (PAB) model.

SUPPLEMENTARY FIGURE 6

Normoxia v SUHx

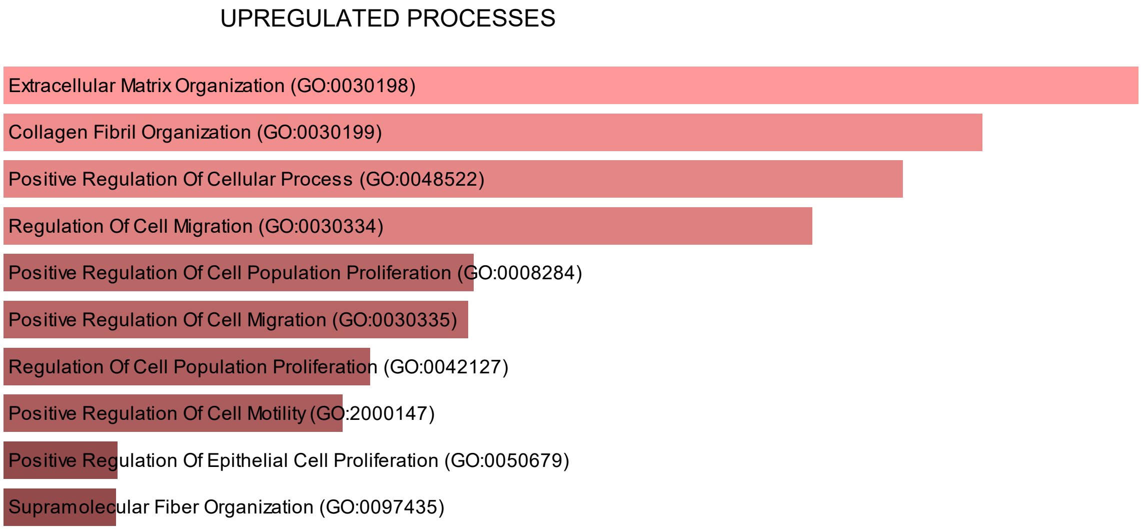

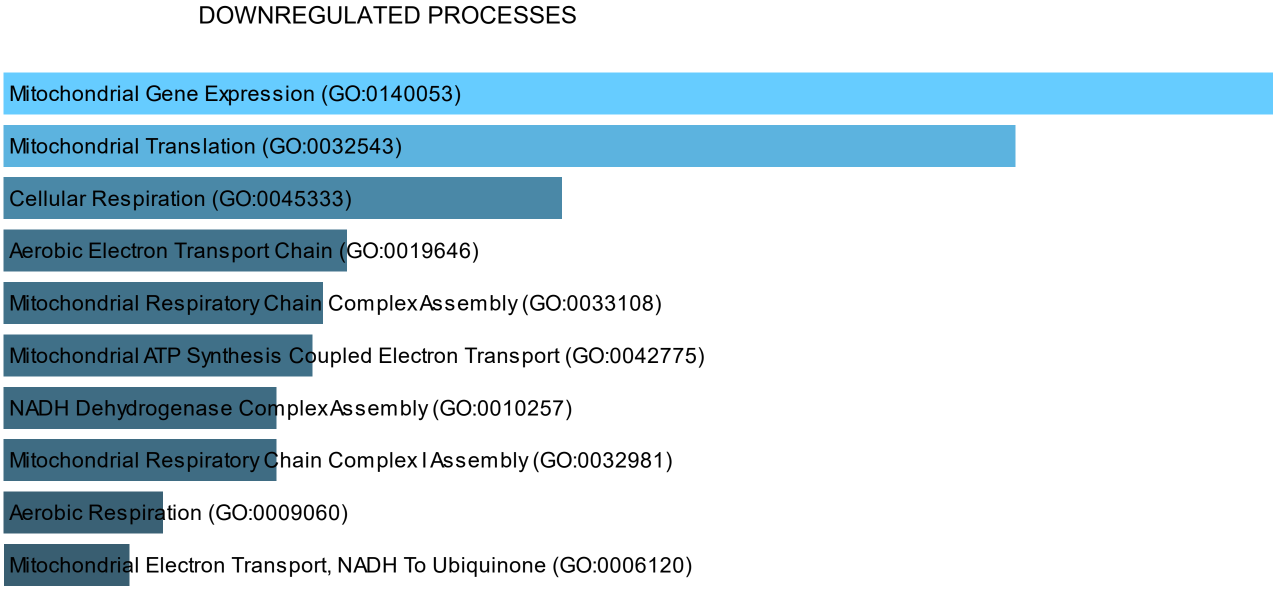

Supplementary Figure 6. Pathway enrichment analysis of DEGs reveal distinctly upregulated (red) and downregulated (blue) pathways that could contribute to RV decompensation in the sugen hypoxia (SUHx) rat model.
